# Physiologic variations of serum tumor markers in gynecological malignancies during pregnancy: a systematic review

**DOI:** 10.1186/1741-7015-10-86

**Published:** 2012-08-08

**Authors:** Sileny N Han, Anouk Lotgerink, Mina Mhallem Gziri, Kristel Van Calsteren, Myriam Hanssens, Frédéric Amant

**Affiliations:** 1 Leuven Cancer Institute, Gynecologic Oncology, University Hospitals Leuven, KU Leuven, Belgium; 2 Department of Obstetrics and Gynecology, Jessa Hospital, Hasselt, Belgium; 3 Foeto-Maternal Unit, University Hospitals Leuven, KU Leuven, Belgium

**Keywords:** anti-Müllerian hormone, CA 125; CA 15-3, cancer, human epididymis secretory protein 4 (HE4), inhibin B, lactate dehydrogenase, pregnancy, squamous-cell carcinoma antigen tumor markers

## Abstract

**Background:**

Recent insights provide support for the treatment of cancer during pregnancy, a coincidence that poses both mother and fetus at risk. Our aim was to critically review studies on the physiologic variations during pregnancy, the most common tumor markers used in diagnosis and follow-up of gynecological cancers.

**Methods:**

We conducted a systematic review of six tumor markers during normal pregnancy: carbohydrate antigen (CA) 15-3 (breast cancer); squamous cell carcinoma antigen (cervical cancer); and CA 125, anti-Müllerian hormone, inhibin B and lactate dehydrogenase (ovarian cancer).

**Results:**

For CA 15-3, 3.3% to 20.0% of all measurements were above the cut-off (maximum 56 U/mL in the third trimester). Squamous cell carcinoma antigen values were above cut-off in 3.1% and 10.5% of the measurements (maximum 4.3 µg/L in the third trimester). Up to 35% of CA 125 levels were above cut-off: levels were highest in the first trimester, with a maximum value up to 550 U/mL. Inhibin B, anti-Müllerian hormone and lactate dehydrogenase levels were not elevated in maternal serum during normal pregnancy.

**Conclusion:**

During normal pregnancy, tumor markers including CA 15.3, squamous cell carcinoma antigen and CA 125 can be elevated; inhibin B, anti-Müllerian hormone and lactate dehydrogenase levels remain below normal cut-off values. Knowledge of physiological variations during pregnancy can be clinically important when managing gynecological cancers in pregnant patients.

## Background

Tumor markers are biochemical substances found in the presence of cancer and produced either by the tumor itself or in response to (para)neoplastic conditions, such as inflammation. Tumor markers can be found in a variety of bodily fluids and tissues and include hormones and several subgroups of (glyco)proteins, such as oncofetal antigens (which are normally expressed during fetal life), enzymes and receptors. They are used for diagnosis, assessment of therapeutic efficacy, and detecting recurrence during follow-up. The most limiting factor in the clinical use of tumor markers is the lack of sensitivity and specificity because the majority of markers are tumor-associated rather than tumor-specific; elevated levels can occur in different types of malignancies as well as in benign and physiological conditions such as pregnancy [1]. Moreover, early diagnosis and treatment of recurrences that are solely detected by the use of tumor marker alone has not shown survival benefit [2].

It is estimated that one in 1,000 to 2,000 pregnant women are diagnosed with an intercurrent malignancy, at an average age of 33 years [3]. Moreover, a slowly rising incidence rate has been observed since the 1960s [4]. Breast cancer, hematological malignancies and cervical cancer are the most commonly encountered malignancies during pregnancy [3]. Pregnancy after oncologic treatment is also becoming more common, mainly due to advances in fertility-sparing treatment and improved prognosis [5]. Diagnosis and treatment of these two types of patients cannot always be extrapolated from the non-pregnant patient; this is also the case when interpreting tumor markers during pregnancy. Unawareness of pregnancy-related physiologic elevations of tumor markers may lead to the search for metastatic disease, using extensive and unnecessary diagnostic examinations that are costly and uncomfortable, and also expose the fetus to avoidable radiation.

At present, the number of studies conducted on serum tumor markers during pregnancy is limited. Our goal is to review existing publications on this topic, and also to provide an easily accessible table of reference values during pregnancy for the most common tumor markers used in cases of gynecological malignancies.

## Methods

**Figure 1:**
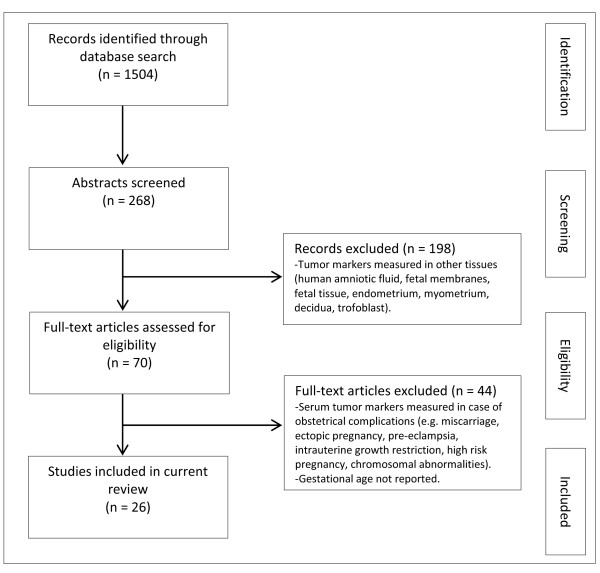
**Methodology for literature review**.

We focused on six tumor markers that are well-established in gynecological cancers and are used for breast cancer (carbohydrate antigen (CA) 15-3), cervical squamous cell cancer (squamous cell carcinoma antigen (SCC)), and ovarian cancer (CA 125 for epithelial ovarian tumors, inhibin B and anti-Müllerian hormone (AMH) for sex cord-stromal tumors, and lactate dehydrogenase (LDH) for germ cell tumors). We conducted a systematic literature search in MEDLINE to identify relevant publications from 1 January 1980 to 31 September 2011 in the English language. Additional publications were identified from the reference lists of relevant articles (Figure 1). The systematic search was conducted using the following medical subject headings (MeSH) terms, words and combinations of words: pregnancy AND CA 15-3, squamous-cell carcinoma antigen, CA 125, inhibin B, anti-Müllerian hormone, lactate dehydrogenase. Two investigators (SH and AL) independently identified potentially relevant articles using the title and the abstract. Eligibility criteria were as follows: firstly, when the maternal serum tumor marker was studied in healthy pregnant women without medical or obstetric confounding conditions, and secondly, if the gestational age was reported by trimester. For inhibin, we excluded older publications that used assays unable to differentiate between dimeric forms and thus were nondiscriminatory between inhibin A and B. Due to the diverse study designs and conditions and use of different assay methods with different intra-and inter-assay coefficients of variation, a meta-analysis was not possible.

α-fetoprotein and the β subunit of human chorionic gonadotropin are both substances that are abundantly present during gestation and have been extensively investigated. Reference values during pregnancy are available in most laboratories, hence we did not include these two markers in our review.

## Results

The database search provided 1,786 articles for the six tumor markers combined. After an initial review of the title and abstract, 54 articles appeared to be relevant and were retrieved to be reviewed in full. Twenty-six studies met our inclusion criteria and were included in the review. Table 1 provides a short summary of the general characteristics of the tumor markers (clinical use, molecular weight and production site). Definitions on the three trimesters of pregnancy varied between publications. The first trimester was defined as the period between the beginning of pregnancy up to 12 to 14 weeks' gestation; the second trimester was defined as the period from the end of the first trimester up to 24 to 28 weeks' gestation, after which began the third trimester until delivery. For each tumor marker, data were extracted from as many studies as possible. These ranges were combined to establish a normal reference range per trimester (Table 2). Cut-off values used in clinical oncology for non-pregnant adults are as stated in the publications and also listed in Table 2.

**Table 1:** Tumor marker characteristics.

Tumor marker	Clinical use in gynecological oncology	Production site in normal adult	Production site during pregnancy	Molecular weight
Carbohydrate antigen 15-3	Breast cancer	Glandular epithelia	Uncertain (maternal mammary gland epithelium? placenta?)	290 kDa
Squamous cell carcinoma antigen	Cervical squamous cell cancer	Squamous epithelia (both benign and malignant)	Uncertain (fetus?)	42 kDa
Carbohydrate antigen 125	Non-mucinous ovarian cancer	Structures derived from the celomic epithelium (such as endocervix, endometrium, and fallopian tube) and in tissues developed from mesothelial cells (such as pleura, pericardium and peritoneum)	Decidua and amnion cells	200 to 250 kDa
Inhibin B	Granulosa cell tumors (some (mucinous) epithelial ovarian tumors)	Granulosa and theca cells (member of the transforming growth factor-β family)	Granulosa and theca cells	Monomer 15 kDa, homodimer 25 kDa
Anti-Müllerian hormone	Granulosa cell tumors	Granulosa cells of ovarian follicles (member of the transforming growth factor-β family)	Sertoli cells of male fetus, for regression of Müllerian ducts	140 kDa
Lactate dehydrogenase	Germ cell tumors	Cell cytoplasm	Cell cytoplasm	140 kDa

**Table 2:** Overview of ranges during pregnancy per tumor marker.

	Normal oncologic cut-off values in non-pregnant women	Trimester 1^a^	Trimester 2	Trimester 3	References
Carbohydrate antigen 15-3^b^	<30 U/mL	5.0 to 39.3	1.0 to 40	7.0 to 56	[7,11,51,52]
		**1.3 to 19.5**	**4.0 to 24.4**	**7.0 to 27**	[6,7]
Squamous cell carcinoma antigen	<2 µg/L	0.3 to 2.9	0.1 to 2.2	0.6 to 4.3	[7]
		**0 to 1.97**	**0.51 to 1.99**	**0 to 2.22**	[6]
Carbohydrate antigen 125	<39 U/mL	3.7 to 550	1 to 166.6	6.1 to 2,419.7	[7,10,11,13,14,17,18]
		**0 to 215.1**	**0 to 308**	**0 to 56.3**	[15,16]
Inhibin B	ng/mL	NA	NA	NA	NA
		**22.06 to 32.94**	**19.88 to 56.12**	**58.62 to 171.38**	[19]
Anti-Müllerian hormone	ng/mL	0.2 to 9.3	0.5 to 4.0	0.2 to 3	[22,23]
		**NA**	**NA**	**NA**	NA
Lactate dehydrogenase	<221 U/L	78 to 433	80 to 447	82 to 524	[25-28]
		**NA**	**NA**	**NA**	NA

NA, not applicable. ^a^The definition of first, second and third trimester is the one used in the study and differs from one study to the other; ^b ^for each parameter the non-pregnant reference value is the mean of all studies, whereas lowest and highest ranges and 2.5 to 97.5 percentiles in the three pregnancy trimesters are presented in the first and the second row, respectively.

### Breast cancer

#### Cancer antigen 15-3

As illustrated in Table 3, CA 15-3 values were described in six publications [6-11], of which two (n = 12 and n = 30) had a longitudinal design [7,11]. Although values largely remained below the cut-off, a significantly increased level was observed during pregnancy in five of the six studies, with the highest levels occurring in the third trimester. In three of the four most recent studies, between 3.3% and 20% of all measurements were found to be above the cut-off value [8-11]. The highest reported CA 15-3 value was 56 U/mL in the third trimester [10].

**Table 3:** Overview of selected studies on carbohydrate antigen 15-3 levels during normal pregnancy.

	Author/year of publication	Study design	Laboratory technique	Number of patients	Cut-off value	Trimester 1	Trimester 2	Trimester 3	Conclusion
1	Touitou 1989 [6]	cross-sectional	IRMA, CIS-Bio International, Gif-Sur-Yvette, France	T1 n = 32; T2 n = 32; T3 n = 36	<25	11.1 ± 4.2 mean ± SD	14.2 ± 5.1	17.0 ± 5.0	None above cut-off value
2	Schlageter 1998 [7]	longitudinal	ELSA-CA 15-3 (CIS-Bio International), Gif-Sur-Yvette, France	n = 12	<30	9.3 ± 4.0 (mean ± SD) 5 to 15 (range)	14.1 ± 4.1 10 to 22.8	16.5 ± 4.1 8.8 to 24.2	None above cut-off value
3	Botsis 1999 [8]	cross-sectional	EIA, Tumor markers CA 153, Abbott AXSYM system, Abbott Park, Il, USA	T1 n = 20; T2 n = 29; T3 n = 26	<33	18.0 (median) 14 to 30 (range)	20 1.0 to 34	22 12 to 41	5%, 10% and 20% above cut-off value, in the 3 trimesters respectively
4	Cheli 1999 [9]	cross-sectional	Bayer Immuno 1 CA 15-3 assay, Tarrytown, New York, USA)	T1 n = 32; T2 n = 5; T3 n = 53	<35	16.76 (mean)	-	20.78	3.3% above cut-off value
5	Bon 2001 [10]	cross-sectional	Enzymun-Test CA 15-3 (Boehringer, Mannheim, Germany)	T1 n = 127; T2 n = 192; T3 n = 47	Not stated	14.0 (median) 5.0 to 32 (range)	15.0 6.0 to 40	26.0 9 to 56	Raised above cut-off value, percentage not stated
6	Ercan 2011 [11]	longitudinal	Modular Analytics E 170 Module (Roche diagnostics), Basel, Switserland.	n = 30	<25	17.5 (median) 7.6 to 39.3 (range)	19.7 10.4 to 39	18.3 7 to 38.6	16% above cut-off value

SD, standard deviation; T, trimester.

### Cervical cancer

#### Squamous cell carcinoma antigen

Physiological circulating levels of SCC throughout gestation have only been reported in two studies to date [6,7]. In 1989, Touitou *et al*. [6] published a cross-sectional study of maternal serum SCC including 32, 32, and 36 women in each of the three pregnancy trimesters, respectively. The observed SCC levels were 0.77 µg/L ± 0.60 (mean ± SD), 1.25 µg/L ± 0.37 and 1.10 µg/l ± 0.56 for the first, second and third trimester, respectively. The SCC levels were significantly higher in the second and third trimesters when compared with the first trimester. The mean concentrations stayed well within the normal range whilst 3.1% of participants had levels exceeding the cut-off value (exact cut-off not stated) [6]. In 1998, Schlageter *et al*. [7] obtained four to nine serum samples from each of 12 healthy pregnant women serially throughout gestation. They also observed higher levels in the third trimester, although mean levels remained below the cut-off throughout the entire pregnancy. SCC concentrations were found to exceed the cut-off value of 1.6 µg/L in 10.5% of samples (range 0.1 to 4.3 µg/L).

### Epithelial ovarian cancer

#### Cancer antigen 125

Although CA 125 is the most studied tumor marker in pregnancy, the different reports are contradictory. We found ten publications [7,10-18], of which four had a longitudinal study design [7,11,15,18]; an overview is shown in Table 4. Elevated levels were found in all ten studies, in up to 35% of the measurements. CA 125 levels were uniformly reported to be highest in the first trimester, with a maximum value up to 550 U/mL [13]. For the second and third trimester, mean maternal CA 125 values were found generally below the cut-off value and remaining below this level until delivery. Nonetheless, four studies found elevated levels up to 73 U/mL in the second trimester [7,10,13,17], and eight studies found elevated levels in the third trimester [7,10,11,13-17], with a maximum level of 2,419.7 U/mL.

**Table 4:** Overview of selected studies on carbohydrate antigen 125 levels during normal pregnancy.

	Author/year of publication	Study design	Laboratory technique	Number of patients	Cut-off value (U/mL)	Trimester 1	Trimester 2	Trimester 3	Conclusion
1	Niloff 1984 [12]	cross-sectional	125I-labeled OC125	N = 101	<65	>65 in 16%	< 65	<65	T1: 16% above cut-off value
2	Haga 1986 [13]	cross-sectional	Centocore Inc., Malvern, PA, USA	T1 n = 29; T2 n = 21; T3 n = 21	<35	85 ± 101 (mean ± SD) 18 to 550 (range)	20 ± 10 10 to 54	25 ± 27 <8 to 140	Raised above cut-off value, percentage not stated
3	Jacobs 1988 [14]	cross-sectional	Abbott Laboratories, Chicago, IL, USA	T1 n = 11; T2 n = 7; T3 n = 8	<35	53.6 (median) 15.6 to 268.3 (range)	18.5 12.0 to 25.1	19.2 16.8 to 43.8	35% above cut-off value
4	Kobayashi 1989 [15]	both cross-sectional and longitudinal	ORIS Industry, Censaclay, France	n = 122	<35	71.7 ± 71.1 (mean ± SD)	19.1 ± 7.0	28.1 ±14.1	Raised above cut-off value, percentage not stated
5	Touitou 1989 [16]	cross-sectional	IRMA, CIS-Bio International, Gif-Sur-Yvette, France	T1 n = 32; T2 n = 32; T3 n= 36	<35	23.7± 13.9 (mean ± SD)	14.8 ± 8.0	22.1 ± 17.1	8% above cut-off value
6	Kenemans 1992 [17]	cross-sectional	Enzymun CA 125 (Boehringer, Mannheim, Germany)	T1 n = 26; T2 n = 20; T3 n = 145	<35	24.4 ± 13.3 (mean ± SD) 54.6 (maximum range)	38.1 ± 47.4 166.6	74.7 ± 273.3 2,419.7	T1: 19% above cut-off value T2: 15% above cut-off value T3: 20% above cut-off value
7	Schlageter 1998 [7]	longitudinal	ELSA-CA 125 (CIS Bio International-Bio International, Gif-Sur-Yvette, France	n = 12	<40	18.7 ± 14.0 (mean ± SD) 6.1 to 41.5 (range)	19.9 ± 12.5 6.5 to 54.3	22.3 ± 13.1 6.1 to 51.3	Raised above cut-off value, percentage not stated
8	Spitzer 1998 [18]	longitudinal	Centocore Inc., Diagnostic division, Malvern, PA, USA	n = 20	<35	33.1 (median) 3.7 to 251.2 (range)	<35	<35	Raised above cut-off value, percentage not stated
9	Bon 2001 [10]	cross-sectional	Enzymun-Test CA 125 (Boehringer, Mannheim, Germany)	T1 n = 127; T2 n = 192; T3 n = 47	Not stated	23 (median) 4 to 108 (range)	14 1 to 73	21 8 to 144	Raised above cut-off value, percentage not stated
10	Ercan 2012 [11]	longitudinal	Modular Analytics E 170 Module (Roche diagnostics, Basel, Switserland	n = 30	<35	19.0 (median) 4.9 to 61 (range)	15.6 4.7 to 32.1	19.6 9.8 to 41.2	4.4% above cut-off value

CA, carbohydrate antigen; ELISA, enzyme-linked immunosorbent assay; IRMA, immunoradiometric assay; SD: standard deviation; T, trimester;

### Sex cord-stromal tumor

#### Inhibin B

To date, two studies have measured inhibin B levels in healthy pregnant women longitudinally during gestation. Petraglia *et al*. [19] followed 13 pregnant women: mean ± SD values showed that serum inhibin B levels during the first (27.50 ± 2.72 ng/L) and second (38.00 ± 9.06 ng/L) trimester were significantly lower than at the third trimester (115.5 ± 28.19 ng/L; *P *<0.001). Values at term were significantly higher than in their control group of non-pregnant women during the early follicular and early luteal phases of the menstrual cycle (*P *<0.01). Fowler *et al*. [20] measured inhibin B in six healthy pregnant women and found that concentrations of inhibin B fell to undetectable concentrations (<12 ng/L) during the first half of pregnancy and only increased slightly in the second half to a maximum concentration of 25 ng/L, which was still well below the normal cut-off level for the non-pregnant premenopausal adult female (and 200-fold lower than inhibin A levels). Wallace *et al*. [21] found undetectable inhibin B levels in maternal serum from 807 pregnancies with 10 to 20 weeks' gestational age.

#### Anti-Müllerian hormone

AMH levels during the three trimesters of pregnancy were published in two articles. La Marca *et al*. [22] conducted a cross-sectional study in 27, 21 and 13 women in the three trimesters respectively, and found that serum AMH values were similar to those of non-pregnant women in the follicular phase, and tended to decrease with progression of the pregnancy. These findings were confirmed by Nelson *et al*. [23] in a prospective longitudinal cohort of 60 pregnant women, they also found normal levels during the first trimester, with a significant decline during the second and third trimester. Lutterodt *et al*. [24] compared first trimester AMH maternal serum levels in relation to the fetal sex (determined by X-Y polymerase chain reaction of fetal tissue after elective termination of pregnancy), and no correlation was found.

### Germ cell tumor

#### Lactate dehydrogenase

During normal uncomplicated pregnancy, reported LDH values all remained below the normal cut-off values [25-28].

## Discussion

Although tumor markers are very commonly used in clinical practice, their relevance and reliability is frequently debated. Tumor markers mainly have a supportive function, even for the routine care of non-pregnant patients. The role of tumor markers is limited in cases of cancer during pregnancy, or pregnancy after cancer, mainly due to their low specificity rate. Elevations are not always correlated with the presence of malignancy but are more often associated with normal physiologic changes of pregnancy. Moreover, obstetrical complications can induce even more variations. For example, elevated CA 125 has been associated with imminent miscarriage [29], and LDH is known to increase in cases of severe preeclampsia and HELLP (hemolysis, elevated liver function tests, low platelets) [26]. Physicians and midwives caring for pregnant women are well aware that the reference ranges of various laboratory values differ during pregnancy [27,30], and this should also be the case with tumor markers in pregnancy (Table 1). Here, we summarize and explain the physiology of elevated levels during pregnancy for CA 15.3, SCC and CA 125. Inhibin-B, AMH and LDH are not elevated during normal pregnancy.

CA 15-3 is a well-characterized immunoassay that allows the detection of the mucin (MUC)-1 antigen. MUC-1 is part of the family of membrane-bound mucins, large glycoproteins, and their expression is frequently elevated in breast cancer cells. Elevated levels can be found in the serum of over 70% of patients with advanced breast cancer [31]. Conflicting data on the possible fetoplacental origin of CA 15-3 are reported. CA 15-3 concentrations in amniotic fluid and/or umbilical cord blood were analyzed and remained very low throughout pregnancy [32-34]; the authors concluded that the combination of an elevated maternal CA 15-3 and low levels in amniotic fluid and umbilical cord blood indicate that the antigen is not produced by the fetus, placenta or decidual tissue, and therefore, could not be considered as an oncofetal antigen [32-35]. However, MUC-1 has been detected in trophoblastic tissue even very early in pregnancy; placental expression increases as the pregnancy progresses and it is highly expressed throughout the third trimester [36,37]. Several authors have hypothesized that CA 15-3 elevations in maternal serum may result from the proliferation of maternal mammary gland epithelium late in pregnancy, with enhanced secretion of mucin, as opposed to placental transfer of the mucin [9,10,35]. Botsis *et al*. [8], and also Ercan *et al*. [11], asserted that CA 15-3 is independent of gestation and remains a reliable tumor marker for breast cancer during pregnancy. This statement is not in accordance with most other studies as found in this review. Although the reported values during pregnancy are only moderately elevated, we believe that caution is warranted, and a higher cut-off value would facilitate interpretation during pregnancy.

Elevated SCC serum levels are found in between 57% and 70% of women with a primary squamous cell carcinoma of the cervix. Elevated levels are also found in between 24% and 53% of patients with squamous cell carcinomas of the head and neck, esophagus, and lung, and also in between 8% and 42% of patients with adenocarcinomas of the ovary and uterus [38]. SCC is probably a marker of cellular differentiation for squamous cells, as the incidence of elevated serum levels is higher in women with grade 1 (78%) and grade 2 (67%) carcinomas than in those with grade 3 tumors (38% )[38]. Sarandakou *et al*. sampled maternal serum, umbilical cord blood and amniotic fluid during delivery of 56 full-termed pregnancies [39]; they found a high incidence of SCC levels above the cut-off value of ≤2.5 µg/L (30% in maternal serum and 75% in umbilical cord blood). The levels found in amniotic fluid were extremely high (median 710 µg/L; range 30 to 7,692 µg/L), which led the authors to conclude that SCC is an oncofetal antigen [39]. The analysis of *in vitro *culture of amnion cells and amniotic membranes revealed no accumulation of SCC in the supernatant, and no mRNA expression of SCC was found in the amnion, the cord or the placenta using a northern blot with a cDNA probe of SCC [40]. Therefore, it is more likely that the fetus, and not the placenta, is the origin of SCC found in amniotic fluid, but this remains to be confirmed.

CA 125 is used for monitoring non-mucinous epithelial ovarian cancer [7,41]. Of patients with ovarian carcinoma, 82% have CA 125 levels >35 U/mL, compared with 1% of apparently healthy non-pregnant individuals. During pregnancy, CA 125 is present in relatively high concentrations in decidual cells, amniotic fluid and amnion cells, and significantly lower levels are found in umbilical cord blood, suggesting that decidua and amnion cells (and not the fetus) produce and secrete CA 125 into the amniotic fluid [39,41,42]. Interestingly, the molecular weight of CA 125 identified in pregnancy was significantly higher than that observed in ovarian cancer, suggesting a different production and/or metabolism of CA 125 glycoprotein for different tissues [35]. The large molecular weight of CA 125 in the fetoplacental unit prevents the passage of the antigen through the basal membranes. Therefore, a large difference exists between amniotic fluid and maternal serum concentrations of CA 125; disruption of the basal membranes can cause a higher permeability from the fetoplacental unit into the maternal circulation [39]. Higher maternal serum CA 125 levels in the first trimester can be explained by the process of trophoblast invasion in the decidua during placentation. Higher levels in the third trimester, and more particularly in the puerperium, can be caused by detachment of the placenta from the uterus, during which time decidual CA 125 might reach the maternal circulation [10].

In persisting adnexal masses during pregnancy, expert ultrasonographic assessment plays a pivotal role in estimating the risk of malignancy, and planning conservative management for an adnexal mass that is probably benign versus surgical treatment during pregnancy for an adnexal mass that has malignant characteristics [43,44]. Ovarian cancer during pregnancy is very rare and has an estimated incidence of 1 in 12,000 to 47,000 pregnancies [43]. When uncertainty remains towards the type of adnexal mass, despite expert evaluation, tumor markers might be important to help formulate the differential diagnosis. From the data presented, it is clear that the usefulness of CA 125 in pregnant women must be carefully considered, as it is evident that maternal serum CA 125 concentrations are influenced by pregnancy, especially during the first trimester. Thus, an adjusted cut-off level should be established in order to interpret CA 125 levels in pregnant patients [35]. Inhibin B and AMH are both serum markers for granulosa cell tumors. Granulosa cell tumors represent about 5% of all primary ovarian neoplasms, and the juvenile type has a higher incidence in children and young women. Currently, there is no evidence-based preference to use inhibin B or AMH as tumor marker in the non-pregnant patient [45]. During pregnancy, an apparent increase in inhibin B immunoreactivity may reflect some cross-reaction with inhibin A. Consequently, it is to be expected that AMH measurements are more reliable during pregnancy than inhibin B.

### Risk of bias

We aimed to minimize risk of bias of individual studies by excluding all studies reporting tumor markers measured in pregnancies with pathology (for example, miscarriage, intrauterine growth restriction, preeclampsia, aneuploidy) and/or without specification of gestational age. Publication bias and selective reporting within studies is not expected for this research area.

### Limitations of the present review and aims for future research

There is no consensus on the clinical benefit of tumor markers and staging procedures. As a result, their practical use differs significantly among centers. Despite this, tumor markers are frequently used in clinical practice. When measured in the pregnant patient, the pregnancy-related physiologic alterations render the interpretation of tumor marker values more difficult. Therefore, we aimed to provide a better knowledge of tumor marker values during pregnancy. The available literature remains inconclusive for several reasons. The majority of studies were cross-sectional and used small cohorts, which may have led to underpowered conclusions. Comparability of study results is further complicated by the different definitions used for the three trimesters of pregnancy and, even more important, by the various types of assays with different intra-and inter-assay coefficients of variation and corresponding different degrees of precision, which were not always mentioned. Confidence intervals and standard deviations were not systematically stated, hence, outliers could not always be excluded. Normal values for pregnant women are still not well established. A longitudinal prospective study with sufficient participants to correct for interpatient heterogeneity would be more suitable to define the 2.5^th ^and 97.5^th ^percentiles for the different tumor markers during pregnancy [1].

Human epididymis secretory protein 4 (HE4, also known as WFDC2) is a new marker for epithelial ovarian carcinoma [46]. HE4 was first proposed as a serum tumor marker for ovarian cancer in 2003 [47]. So far, its value as an additional marker alongside CA 125 is still under debate [48,49]. Interestingly, HE4 has an increased performance in the premenopausal group, mainly because, unlike CA 125, it is not overexpressed in cases of endometriosis [50]. The expression of HE4 during normal pregnancy deserves further investigation.

## Conclusion

Based on this review, we can conclude that CA 125 values can be raised during pregnancy and both CA 15.3 and SCC levels generally remain below the cut-off values, although higher levels are not uncommon. Inhibin B, AMH and LDH levels are not elevated in maternal serum during normal pregnancy. Despite its aforementioned limitations, the reference table we have assembled provides a quick reference for gynecological tumor markers during pregnancy.

## Abbreviations

AFP: α-fetoprotein; AMH: anti-Müllerian hormone; CA: cancer antigen; HE4: human epididymis secretory protein 4; LDH: lactate dehydrogenase; MUC-1: mucin-1; SCC: squamous-cell carcinoma antigen; SD: standard deviation.

## Competing interests

The authors declare that they have no competing interests.

## Authors' contributions

SH developed the concept for the review with MH and FA. Literature research, data interpretation and first manuscript draft were done by SH and AL. SH, AL, MMG, KVC, MH and FA critically revised the content. All authors read and approved the final version.

## Pre-publication history

The pre-publication history for this paper can be accessed here:

http://www.biomedcentral.com/1741-7015/10/86/prepub
